# Notes and Comments

**Published:** 1897-09

**Authors:** 


### Notes and Comments.[1]

International Dental Gold-Mining Company.—A communication from Dr. J. C. Townsend, formerly of Philadelphia, now of Colorado Springs, announces the formation of the above-named company. Dr. Townsend went to Colorado several years ago to regain his health. He is now a member of the Board of Trade and the Mining Exchange of Colorado Springs, and states that he will be pleased to serve his former professional friends in the East.

A Help for Insomnia.—It is a well-known fact that a "drowsy" feeling usually follows a hearty dinner. This is accounted for by so large a part of the blood of the system being called to the stomach to aid in digestion, leaving the brain poorly supplied. The writer has for a number of years partaken of a light but wholesome lunch before retiring, and after a day filled with various duties, and the evening occupied with literary and other labors, he always finds ready and restful sleep following the midnight lunch.

[1] The assistant editor solicits contributions for this department,—new methods, new remedies and formulas, or any short practical note which may prove of value to the practitioner or student. Address 1718 Walnut Street, Philadelphia.

